# Computed tomographic colonography in the evaluation of a case of giant colonic diverticulum

**DOI:** 10.1259/bjrcr.20160101

**Published:** 2016-12-23

**Authors:** Aldo Carnevale, Matteo Bassi, Zairo Ferrante, Roberto Rizzati, Giorgio Benea, Melchiore Giganti

**Affiliations:** ^1^Scuola di Specializzazione in Radiodiagnostica, University of Ferrara, Ferrara, Italy; ^2^Department of Radiology, Arcispedale Sant’Anna, Ferrara, Italy

## Abstract

The aim of this article was to present our experience with CT-colonography evaluation of a case of giant colonic diverticulum. Despite the high prevalence of diverticular disease in the Western world, giant colonic diverticula are rare entities, with fewer than 200 cases reported in literature.

## Clinical presentation

A 63-year-old male presented to the radiology department with a history of abdominal pain over the hypogastrium and associated bowel disturbance worsening in the last few months.

He received a positive faecal occult blood test for screening. An optical colonoscopy was performed, but turned out to be incomplete for the presence of a stricture at the level of the sigma, reporting the presence of numerous small diverticula in that tract. Routine blood tests revealed a mild microcytic hypochromic anaemia.

## Investigations

**Figure 1.**
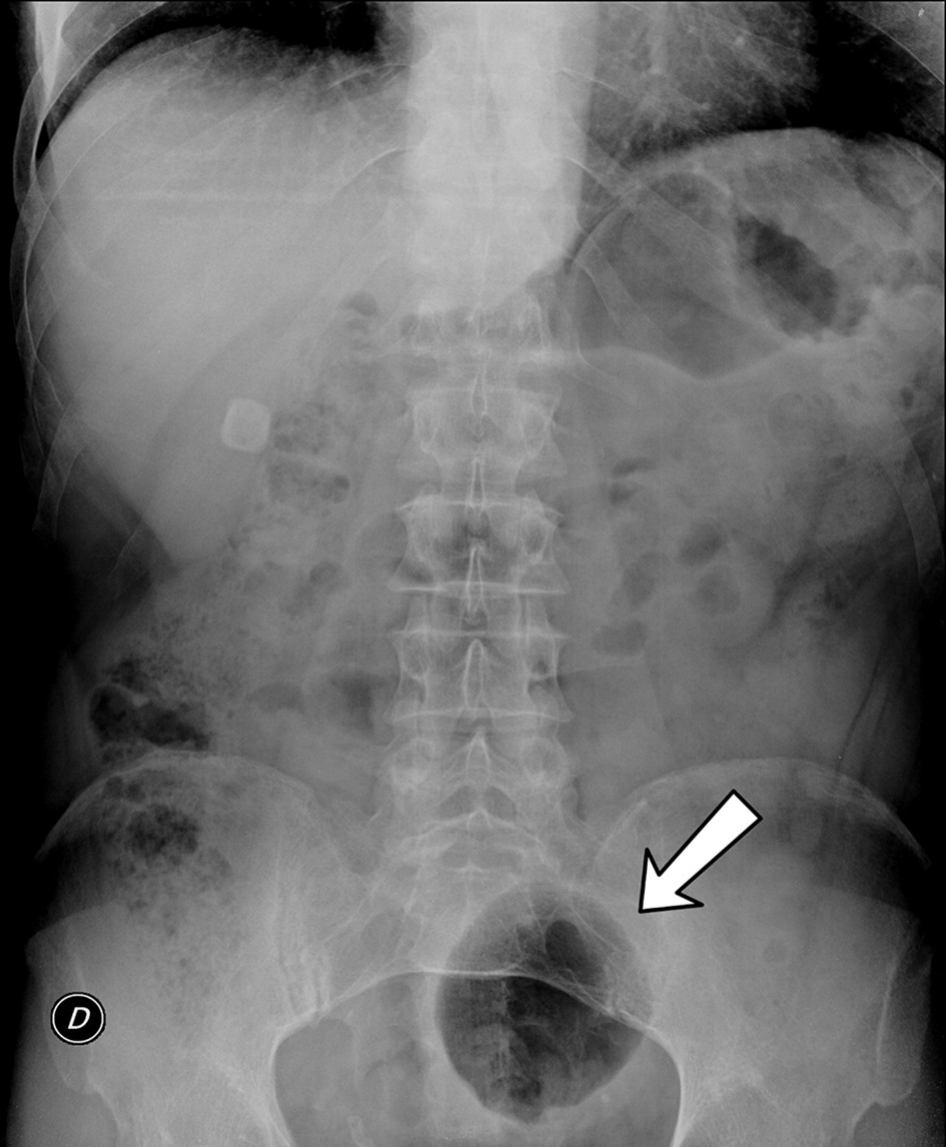
Abdominal X-rays showing a large gas-filled cavity in the lower abdomen (arrow).

Abdominal series consisting of posterioranteriorupright view and supine lateral view radiographs of the abdomen showed a large well-defined oval air-filled formation in the lower abdominal cavity (Figure 1). The remainder of bowel gas pattern was unremarkable. No evidence of free intraperitoneal air.

**Figure 2.**
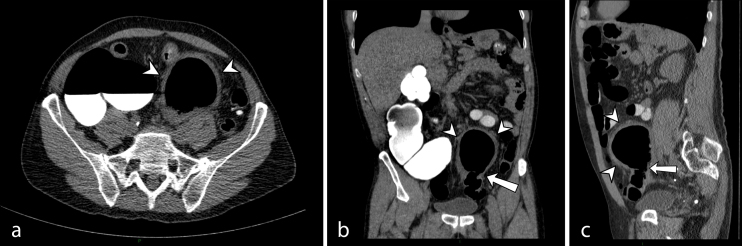
(a) Axial, (b) coronal and (c) sagittal sections of abdomen and pelvis computed tomography colonography demonstrating a large, midline cystic-appearing structure with surrounding inflammatory stranding (arrowheads). Communication to the sigmoid colon can be seen (arrow).

According to Italian guidelines for colorectal cancer screening (patients with positive faecal occult blood test after incomplete optical colonoscopy), a CT colonography (CTC) (64-row CT scan; VCT, General Electric Healthcare, Waukesha, WI, USA) was performed: axial scans with sagittal and coronal reconstructed images showed a thick-walled, 7 cm cystic mass appeared to arise from the sigmoid colon (Figure 2).

Circumferential thickening of sigmoid colon walls was noted, consistent with inflammatory changes in severe diverticular desease. Indeed, multiple small diverticula were reported originating from colonic walls, as well as surrounding mesenteric fat stranding.

**Figure 3.**
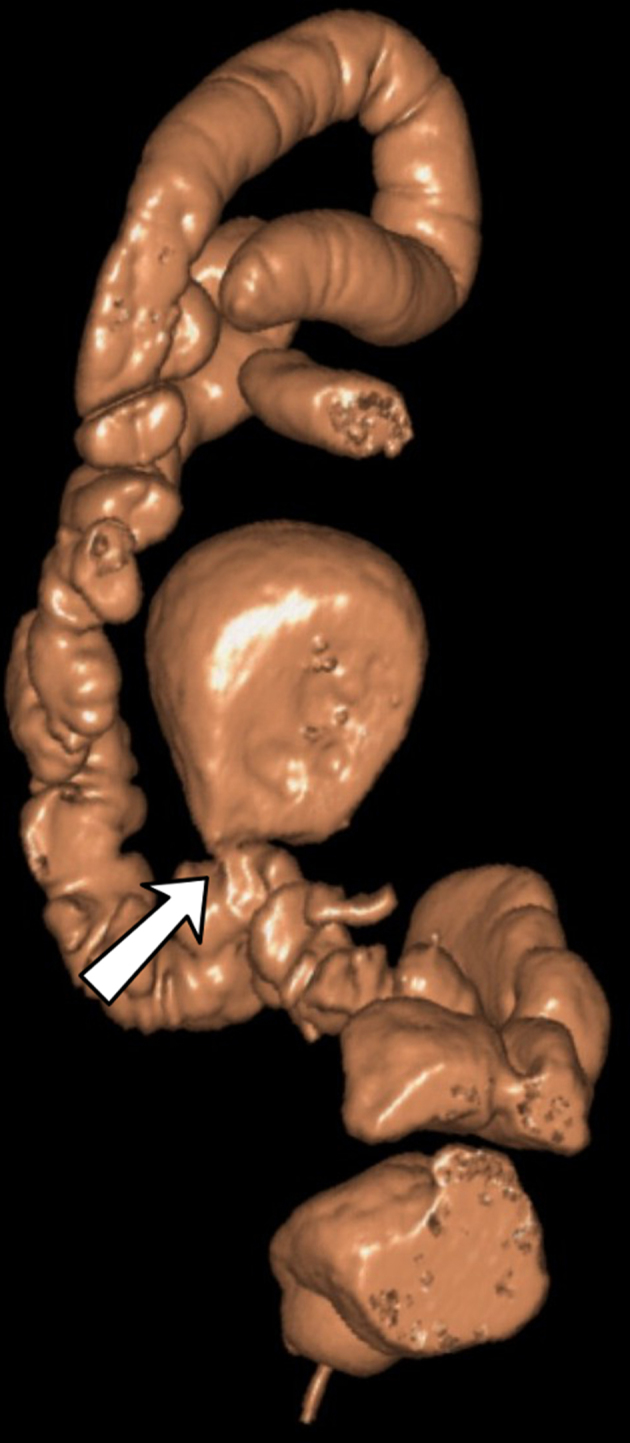
CT 3D colon map, supine position, showing a thin connection of giant colonic diverticulum to sigmoid colon lumen (arrow).

3D reconstructions on CTC dataset were obtained (Figure 3), showing a thin connection of the cystic mass to the bowel lumen. All these findings were suggestive of a giant colonic diverticulum (GCD).

Two days later, for the purpose of a complete preoperative study, a CT after intravenous injection of non-ionic iodinated contrast material (Iomeron® 400, Bracco SpA, Milan, Italy) was performed; the wall of the formation showed a weak delayed enhancement.

## Differential diagnosis

The differential diagnosis includes sigmoid or cecal volvolus, pneumatosis cystoides intestinalis, intestinal duplication cyst, giant Meckel’s diverticulum and vesicoenteric fistula.

Exclusion of alternative diagnoses can be reasonably made by CT scan, observing location and features of the cystic structure, first of all demonstrating its neck, a thin connection to the colonic lumen, well depicted by CTC.

Swirling of the bowel and its mesentery is a typical CT finding in volvulus of the gastrointestinal tract, resulting in dilatation of intestinal loops caused by mechanic obstruction.

Pneumatosis cystoides intestinalis is an uncommon entity, in most cases an incidental finding. It represents an idiopathic cystic gas collection in the intestinal subserosa or mucosa.^1^

Enteric duplication cysts are unusual congenital abnormalities. They can occur anywhere along the digestive tract on the mesenteric side, involving more often the small intestine. They present as a simple fluid cyst with mass effect extending into the bowel lumen. Most duplication cysts manifest early, during the first year of life.

Meckel’s diverticulum is the most frequent intestinal congenital anomaly, usually found within 100 cm of the ileocecal valve on the antimesenteric border of the ileum.

Fistulous connections between the bladder and small or large bowel most often develop in the setting of inflammatory or neoplastic gastrointestinal or genitourinary diseases, leading to gas entering the bladder lumen.

## Treatment, outcome and follow-up

**Figure 4.**
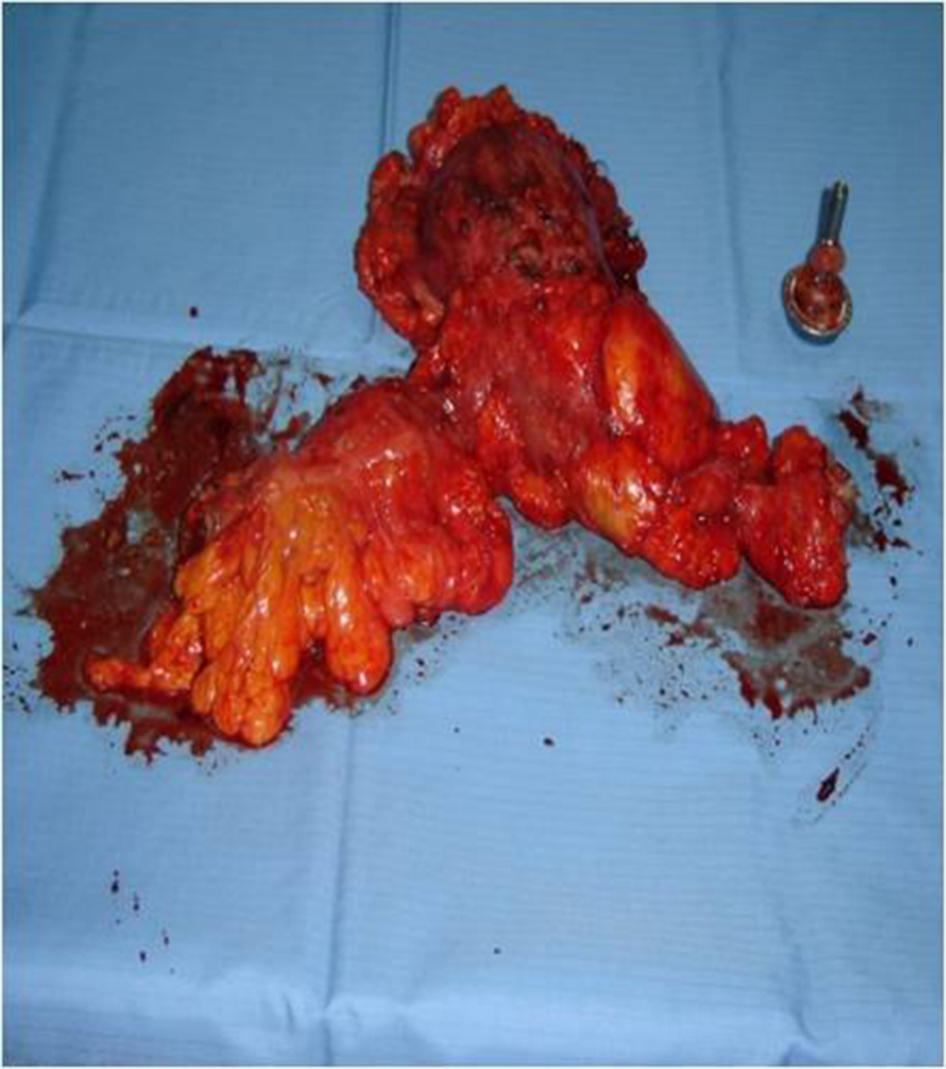
Operative specimen.

**Figure 5.**
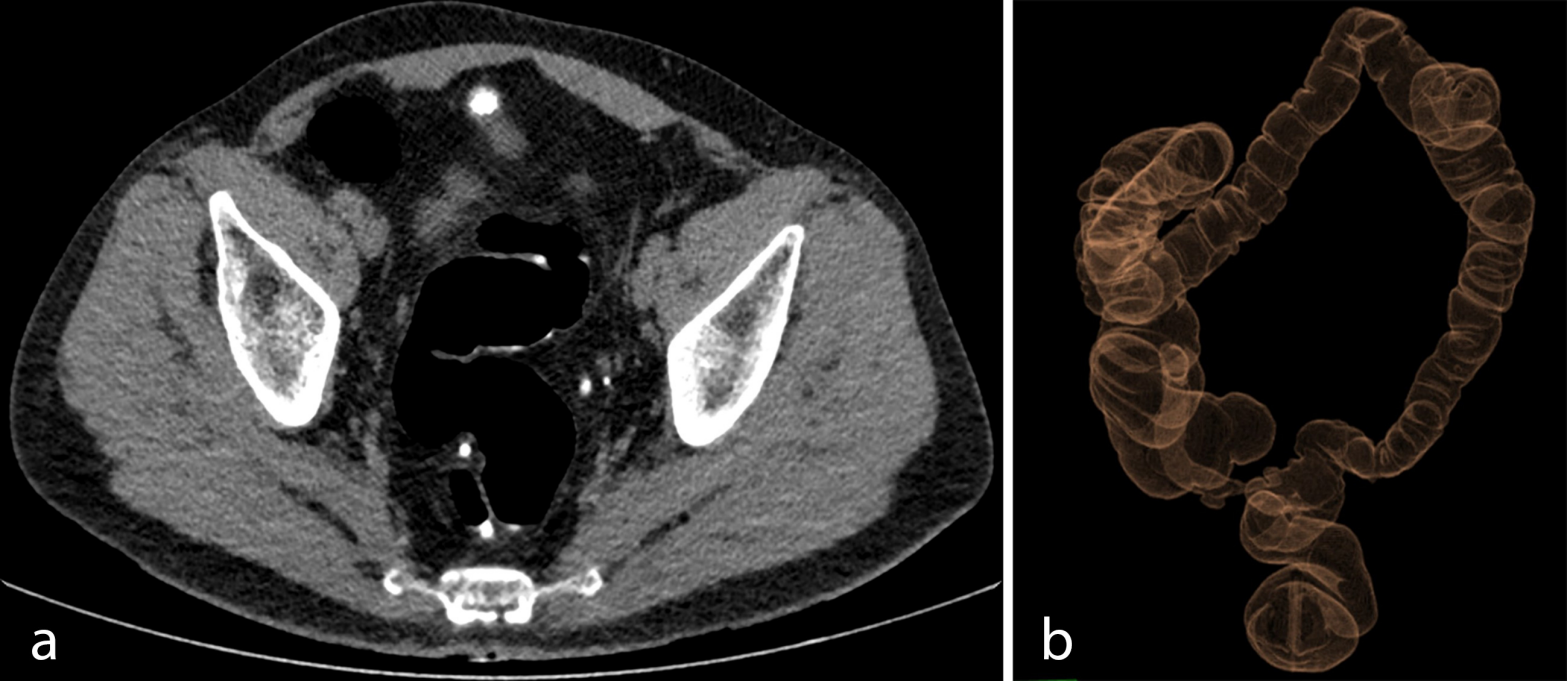
CTC images obtained after surgical intervention: (a) axial section on anastomosis site and (b) CT 3D colon map.

The patient underwent laparotomic sigmoid colectomy, with excision of the giant diverticulum and primary anastomosis, where a large air-filled cyst was found developing from the mesenteric border of the proximal sigmoid colon (Figure 4). Six weeks later, a new CTC study was obtained (Figure 5).

## Discussion

Despite the high prevalence of diverticular desease in the Western world, giant colonic diverticula are rare entities, with fewer than 200 cases reported in literature.

By definition, they are outpouchings of the colonic wall greater than 4 cm in size, the diameter can vary, with the largest described being 40 cm.

Approximately 90% of them develop from the sigmoid colon. They might be isolated, but in 85% of the cases, GCDs are associated with concomitant diverticulosis or diverticular disease.^2^ The majority of GCD presents after the 6th decade of life.

Clinical manifestation is variable, ranging from asymptomatic palpable abdominal mass to acute abdomen. Perforation and abscess formation are the most common encountered complications;^3^ it remains difficult to establish any relation of cause and effect between GCD and cancer development.

McNutt et al^4^ classified GCD in three histological and pathogenetic subtypes. Type 1 diverticula (22% of the cases, according to Steenvoorde et al^5^) are pulsion diverticula, which gradually enlarge, representing outpouchings of the colonic mucosa and submucosa through the muscular layers of the wall, where the vessels pierce the muscularis (pseudodiverticula such as common colonic diverticula).

Type 2 diverticula (inflammatory diverticula, 66% of the cases^5^) are secondary to a subserosal perforation, leading to a walled off abscess cavity communicating with the bowel lumen and gradually enlarging. Their wall is composed of fibrous scar tissue, without a normal intestinal layer.

Type 3 (true diverticula, 12% of the cases^5^) most likely have a congenital origin and are formed by all the bowel layers.

The diagnosis of GCD is often challenging, because patient symptoms and laboratory findings are unspecific and overlap with other gastroenterological entities; it is necessarily confirmed by radiologic studies. A plain abdominal radiograph usually demonstrates a large gas-filled cyst (balloon sign).^6^

Ultrasound examination does not seem to be the radiological examination of choice for detecting a GCD: no significant abnormalities are usually detected.

Diagnostic colonoscopy is frequently non-contributory: it is considered that the ostium of diverticular neck might be too tight to allow visualisation of the interior aspect.^5^ A contrast enema can demonstrate the communication of the diverticulum to the bowel lumen in almost 70% of cases.

Multidetector CT is the preferred imaging technique for GCD evaluation, as it is for other complications of colonic diverticular disease. CT demonstrates a smooth-walled gas-containing structure, rarely in association to marginal calcification. An air-fluid level can be sometimes observed. Coronal and sagittal multiplanar reformatted images are important for identifying the neck of the GCD, that is its connection to the bowel lumen; this finding is essential for a correct diagnosis.^7^

The wall of the cyst is usually smooth and regular, but if irregular or lobulated, then the possibility of an additional inflammatory or neoplastic process should be considered.

The investigation of diverticular disease is a clear clinical situation where CTC can be safely proposed:^8^ it may be useful in GCD evaluation, but its role is difficult to establish, in relation to the rarity of this condition. In our single experience, no complications were encountered during the procedure; CTC offered a valid instrument to study the diverticulum’s anatomical features, providing as well a panoramic view of colon and remaining abdominal organs. Particularly, this technique proved to be useful to demonstrate the diverticular neck, well depicted thanks to colon distension also in 3D reconstructed images.

The prevalence of complications in GCD and their potential severity impose surgical treatment, even including asymptomatic GCD discovered fortuitously. This attitude can be supported by the low morbidity and mortality of elective colonic resections.^9^

## Learning points

1. By definition, giant colonic diverticula are outpouchings of the colonic wall greater than 4 cm in size.
2. They are rare entities, in most cases associated to diverticular desease.
3. The diagnosis is often challenging, because patient symptoms and laboratory findings are unspecific and overlap with other gastroenterological entities.
4. Multi-detector computed tomography is the preferred imaging technique for their evaluation, essential for identifying the connection to bowel lumen.

## Consent

Written informed consent for the case to be published (incl. images, case history and data) was obtained from the patient(s) for publication of this case report, including accompanying images.
